# Supplementary material for: Supervised multiple kernel learning approaches for multi-omics data integration
Source: BioData Min. 2024 Nov 23;17:53. doi: 10.1186/s13040-024-00406-9 (PMC11585117; doi:10.1186/s13040-024-00406-9)
Supplement: Supplementary file 1 — Supplementary Material 1. [file 13040_2024_406_MOESM1_ESM.pdf]

Supplementary Information

Mitja Briscik, Gabriele Tazza,

László Vidács, Marie-Agnès Dillies, Sébastien Déjean

October 31, 2024

1 Supplementary Tables

1.1 Integration modes

In this section we present the results of the comparison between the different integration modes of the deep learning architectures.

	ROSMAP		
Algorithm	ACC	AUC	F1
Deep MKL (concat)	$\mathbf{0.747 \pm 0.018}$	0.810 ± 0.021	0.762 ± 0.014
Deep MKL (sum)	0.745 ± 0.020	0.805 ± 0.020	0.762 ± 0.014
Deep MKL (weighted sum)	0.715 ± 0.028	0.800 ± 0.021	0.721 ± 0.027
Cross-Modal Deep MKL (concat)	$\mathbf{0.732 \pm 0.020}$	0.808 ± 0.018	0.751 ± 0.025
Cross-Modal Deep MKL (sum)	0.726 ± 0.021	0.809 ± 0.018	0.739 ± 0.043
Cross-Modal Deep MKL (weighted sum)	0.730 ± 0.025	0.802 ± 0.020	0.746 ± 0.039

Table 1: Metrics average and standard deviation over 5 random test splits for the performance evaluation on ROSMAP dataset.

Algorithm	BRCA		
	ACC	F1_weighted	F1_macro
Deep MKL (concat)	0.835 ± 0.016	0.801 ± 0.021	0.840 ± 0.020
Deep MKL (sum)	$\mathbf{0.836 \pm 0.029}$	0.812 ± 0.036	0.842 ± 0.029
Deep MKL (weighted sum)	0.827 ± 0.014	0.803 ± 0.015	0.831 ± 0.013
Cross-Modal Deep MKL (concat)	$\mathbf{0.828 \pm 0.015}$	0.802 ± 0.018	0.832 ± 0.021
Cross-Modal Deep MKL (sum)	0.822 ± 0.027	0.786 ± 0.037	0.824 ± 0.030
Cross-Modal Deep MKL (weighted sum)	0.829 ± 0.017	0.802 ± 0.022	0.834 ± 0.015

Table 2: Metrics average and standard deviation over 5 random test splits for the performance evaluation on BRCA dataset.

Algorithm	LGG		
	ACC	AUC	F1
Deep MKL (concat)	0.680 ± 0.028	0.763 ± 0.025	0.688 ± 0.019
Deep MKL (sum)	0.680 ± 0.018	0.770 ± 0.015	0.683 ± 0.024
Deep MKL (weighted sum)	$\mathbf{0.687 \pm 0.011}$	0.765 ± 0.025	0.684 ± 0.031
Cross-Modal Deep MKL (concat)	0.693 ± 0.012	0.758 ± 0.024	0.678 ± 0.023
Cross-Modal Deep MKL (sum)	0.695 ± 0.022	0.763 ± 0.023	0.678 ± 0.028
Cross-Modal Deep MKL (weighted sum)	$\mathbf{0.700 \pm 0.020}$	0.768 ± 0.026	0.695 ± 0.032

Table 3: Metrics average and standard deviation over 5 random test splits for the performance evaluation on LGG dataset.

	KIPAN		
Algorithm	ACC	F1_weighted	F1_macro
Deep MKL (concat)	0.951 ± 0.010	0.945 ± 0.018	0.951 ± 0.0101
Deep MKL (sum)	0.956 ± 0.008	0.950 ± 0.019	0.956 ± 0.008
Deep MKL (weighted sum)	$\mathbf{0.958 \pm 0.011}$	0.954 ± 0.018	0.958 ± 0.011
Cross-Modal Deep MKL (concat)	0.957 ± 0.010	0.948 ± 0.021	0.957 ± 0.010
Cross-Modal Deep MKL (sum)	0.956 ± 0.009	0.950 ± 0.019	0.956 ± 0.009
Cross-Modal Deep MKL (weighted sum)	$\mathbf{0.958 \pm 0.009}$	0.952 ± 0.014	0.958 ± 0.009

Table 4: Metrics average and standard deviation over 5 random test splits for the performance evaluation on KIPAN dataset.

1.2 DeepMKL configurations

This section presents the results of a comparative analysis between different DeepMKL configurations. Specifically, we want to explore the effect of the neural network architecture's depth and width on the classification performance. We conducted these experiments using DeepMKL (weighted sum) on the BRCA and ROSMAP datasets. We started with the same configuration choices, i.e. [200,200,100] for ROSMAP and [400,400,200] for BRCA, used in MOGONET, which are also the ones reported in the Results Section of the article. Then, we explored different configurations for depth and width.
As shown in Tables 5, 6, we tested three configurations for DeepMKL with two, three, and four layers. For each of these DeepMKL architectures, we tested three configurations with different numbers of layers, doubling and halving the number of neurons w.r.t our baseline. The results show that for the BRCA, DeepMKL is robust w.r.t differences in depth and width. For ROSMAP, the effect of varying the number of neurons is more clear. The three configurations with fewer neurons have worse performances in the case of DeepMKL with two, three, and four layers. While the ones with the larges number of neurons obtain the best performances. Similarly to the BRCA case, the DeepMKL for ROSMAP architecture seems robust w.r.t. the variation in the number of layers.

	BRCA		
DeepMKL Configuration	ACC	F1_weighted	F1_macro
[200, 100]	0.835 ± 0.020	0.841 ± 0.021	0.813 ± 0.024
[400, 200]	0.837 ± 0.016	0.843 ± 0.016	0.813 ± 0.022
[800, 400]	0.826 ± 0.028	0.831 ± 0.028	0.804 ± 0.030
[200, 200, 100]	0.833 ± 0.018	0.838 ± 0.019	0.808 ± 0.026
[400, 400, 200] (baseline)	0.827 ± 0.014	0.831 ± 0.013	0.803 ± 0.015
[800, 800, 400]	0.832 ± 0.027	0.838 ± 0.028	0.811 ± 0.028
[200, 200, 200, 100]	0.834 ± 0.016	0.838 ± 0.016	0.810 ± 0.021
[400, 400, 400, 200]	0.842 ± 0.019	0.849 ± 0.018	0.823 ± 0.014
[800, 800, 800, 400]	0.831 ± 0.025	0.837 ± 0.024	0.808 ± 0.024

Table 5: Comparative study for different width and depth of the architecture - BRCA dataset.

DeepMKL Configuration	ROSMAP		
	ACC	AUC	F1
$[100, 50]$	0.689 ± 0.031	0.768 ± 0.028	0.697 ± 0.033
$[200, 100]$	0.717 ± 0.015	0.801 ± 0.021	0.719 ± 0.023
$[400, 200]$	0.736 ± 0.010	0.805 ± 0.015	0.746 ± 0.014
$[100, 100, 50]$	0.704 ± 0.022	0.774 ± 0.018	0.687 ± 0.038
$[200, 200, 100]$ (baseline)	0.715 ± 0.028	0.800 ± 0.021	0.721 ± 0.027
$[400, 400, 200]$	0.726 ± 0.013	0.806 ± 0.022	0.747 ± 0.022
$[100, 100, 100, 50]$	0.649 ± 0.070	0.739 ± 0.073	0.690 ± 0.045
$[200, 200, 200, 100]$	0.724 ± 0.016	0.808 ± 0.016	0.732 ± 0.015
$[400, 400, 400, 200]$	0.726 ± 0.025	0.802 ± 0.014	0.747 ± 0.030

Table 6: Comparative study for different width and depth of the architecture - ROSMAP dataset.
